# Isolated Penile Calciphylaxis Diagnosed by Ultrasound Imaging in a New Dialysis Patient: A Case Report

**DOI:** 10.1177/20543581211025846

**Published:** 2021-07-26

**Authors:** Wryan Helmeczi, Tyler Pitre, Emma Hudson, Suhas Mondhe, Kevin Burns

**Affiliations:** 1 Department of Internal Medicine, University of Ottawa, ON, Canada; 2 Department of Internal Medicine, McMaster University, Hamilton, ON, Canada; 3 Department of Family Medicine, Queen’s University, Kingston, ON, Canada; 4 Department of Nephrology, University of Ottawa, ON, Canada

**Keywords:** calciphylaxis, penile, diagnosis, ultrasound, radiograph

## Abstract

**Rationale:**

The recognition of calciphylaxis often eludes practitioners because of its multiple ambiguous presentations. It classically targets areas of the body dense with adipose tissue. A heightened suspicion for the disorder is therefore required in the case of penile calciphylaxis, given its unconventional location. The diagnosis of calciphylaxis is also challenging as the gold standard for diagnosis is biopsy which can often yield equivocal results. Unfortunately, in penile calciphylaxis, the utility of biopsies is further debated due to their potential to precipitate new lesions and their decreased sensitivity due to the limited depth of tissue that can be sampled. For these reasons, it is important that practitioners recognize other accessible and accurate investigative tools which can aid in their diagnosis.

**Presenting concerns of the patient:**

We present the case of a 49-year-old man who presented to the emergency room with penile pain in the context of known chronic kidney disease secondary to diabetic nephropathy. The pain had been present for about a week, was exquisitely tender, and was initially associated with a faint violaceous lesion. This gentleman had just recently initiated peritoneal dialysis and had no other lesions on his body.

**Diagnosis:**

His pain was determined by ultrasound and plain radiograph to be secondary to calciphylaxis after two biopsies were nondiagnostic.

**Interventions:**

The patient had already made changes to his diet to reduce phosphate and calcium intake, and had been on phosphate-lowering therapy with both calcium and phosphate being within their respective target range. Following his diagnosis, this patient was promptly converted from peritoneal dialysis to hemodialysis with sodium thiosulphate and initiated hyperbaric oxygen therapy. This patient continues to be followed by nephrology and urology specialists.

## Abrégé

**Outcomes:**

At the time of publication there has been no amputation of the appendage although there has been presumably permanent loss of function.

**Teaching points:**

This case should reinforce the high index of suspicion needed to make a timely diagnosis of calciphylaxis, and the broad variety of ways in which the disorder may present. The findings advocate for the utility of alternative diagnostic modalities, including imaging with plain radiograph and ultrasound, for calciphylaxis diagnosis.

**Justification:**

Les multiples présentations cliniques de la calciphylaxie, une affection qui cible habituellement des régions du corps denses en tissu adipeux, font en sorte que sa détection échappe fréquemment aux praticiens. La calciphylaxie pénienne, en raison de son emplacement inhabituel, devrait faire l’objet d’une suspicion clinique accrue. La calciphylaxie est en outre difficile à diagnostiquer puisque la biopsie, l’étalon-or pour sa détection, mène souvent à des résultats équivoques. Dans le cas de la calciphylaxie pénienne, la pertinence de la biopsie fait d’autant plus débat puisqu’elle est susceptible de précipiter de nouvelles lésions et qu’elle présente une sensibilité réduite compte tenu de la profondeur limitée des tissus pouvant être obtenus. Il est donc important que les praticiens connaissent d’autres outils d’investigation accessibles et plus précis pouvant les aider dans leur diagnostic.

**Présentation du cas:**

Nous présentons le cas d’un homme de 49 ans s’étant présenté aux urgences avec une douleur au pénis. Le patient était connu pour une insuffisance rénale chronique découlant d’une néphropathie diabétique. La douleur, initialement associée à une lésion légèrement violacée, était présente depuis environ une semaine et le membre était extrêmement sensible. Le patient venait tout juste d’amorcer des traitements de dialyze péritonéale et ne présentait aucune autre lésion sur le corps.

**Diagnostic:**

Après deux biopsies non diagnostiques, une échographie et une radiographie standard ont permis d’associer la douleur à la calciphylaxie.

**Interventions:**

Le patient avait déjà apporté des changements à son alimentation afin de réduire son apport en phosphore et en calcium, et recevait déjà un traitement de chélateur de phosphate. Les taux de calcium et de phosphore se situaient dans leur plage cible respective. À la suite du diagnostic, le patient est rapidement passé de la dialyze péritonéale à l’hémodialyse, a été traité avec du thiosulfate de sodium et a entrepris une oxygénothérapie hyperbare. Le patient continue d’être suivi par des spécialistes en néphrologie et en urologie.

**Résultats:**

Au moment de la publication, le patient n’avait toujours pas subi d’amputation, malgré une vraisemblable perte de fonction permanente.

**Enseignements tirés:**

Ce cas devrait renforcer l’indice élevé de suspicion qui est nécessaire pour poser rapidement un diagnostic de calciphylaxie, en plus de montrer les très nombreuses présentations cliniques de ce trouble. Ces résultats plaident en faveur de modalités alternatives pour le diagnostic de la calciphylaxie, notamment le recours à l’échographie et à la radiographie standard.

## Introduction

Calciphylaxis most often occurs in the context of end-stage renal disease (ESRD) with a median onset of presentation 925 days after the initiation of hemodialysis.^1^ In dialysis patients, calciphylaxis is more likely to present in a central distribution affecting predominantly the abdomen and thighs.^1-3^ This patient presented with calciphylaxis only affecting the glans of the penis, a rare but well-described phenomenon in literature.^4^ Biopsies are the gold standard for diagnosis of calciphylaxis but may theoretically lead to another foci of disease, as trauma is related to the precipitation of new lesions.^1-3,5^ This patient had 2 biopsies done which were nondiagnostic. Biopsies are often contraindicated or inconclusive, and clinicians are left to rely on alternative means to establish the diagnosis of calciphylaxis.^1,4^ Bone scintigraphy is the imaging modality of choice when diagnosing calciphylaxis, but the utility is unknown in penile disease.^6^ Calciphylaxis has historically been underrecognized and requires a high index of suspicion and prompt diagnosis for meaningful therapy and preservation of tissue.^1,2,4^ This case study reinforces the vigilance required from clinicians to promptly recognize and treat this entity.

## Presenting Concerns

A 49-year-old Caucasian man presented to the emergency room with a 5-day history of sharp and constant penile pain of sudden onset. The pain was moderate at baseline but became excruciating with any manipulation of the area. The patient was unaware if there was any associated lesion prior to arrival because he did not check the area often due to his erectile dysfunction.

## Clinical Findings

Relevant past medical history included chronic kidney disease (CKD) secondary to diabetic nephropathy (DN). He had no history of skin cancer or rheumatic conditions. He denied the use of any medication strongly associated with a fixed drug eruption. Numerous complications from diabetes had previously developed including retinopathy, peripheral neuropathy, nephropathy, erectile dysfunction, and coronary artery disease.

This patient had proteinuria as early as 2012, when his albumin-creatinine ratio (ACR) was 50 g/mol. His blood pressure was well controlled with telmisartan/hydrochlorothiazide. His diabetes was suboptimally controlled for several years. In 2016, his HbA1c was 12.4% which reached target by 2020 with progression of his renal disease, dietary modification, linagliptin, and basal insulin glargine. He was noted to have increased creatinine of 148 µmol/L (eGFR 50 mL/min/m^2^) in 2016 which steadily progressed, and in July 2020, his creatinine had increased to 650 µmol/L. In August 2020, he began peritoneal dialysis (PD) due to refractory volume overload.

Laboratory indicators of chronic kidney disease bone-mineral disorder (CKD-BMD) were detected as early as October 2019. He was managed with calcitriol, calcium carbonate, and a vegan diet. Prior to his first admission in the summer of 2020, his calcium was 2.32 mmol/L, phosphate was 1.75 mmol/L, and PTH was 3.0 pmol/L. He was never found to be hypercalcemic but had persistent hyperphosphatemia greater than 2.00 mmol/L for three months earlier in 2020. This patient never required a calcimimetic or bisphosphonate.

This patient was not known to use alcohol, tobacco products, cannabis, or any illicit substance. They were obese, worked a sedentary job, and recently began a vegan diet but previously consumed high quantities of processed foods. There was no family history of kidney disease. His father had a history of psoriasis, coronary artery disease with a myocardial infarction at age 54, and passed away from cardiac disease at age 56.

**Figure 1.**
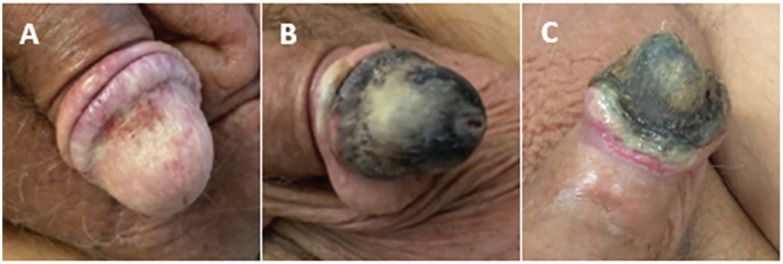
Depicts the evolution of the penile lesion on initial presentation (subfigure A), 2 weeks after initial presentation prior to any therapy (subfigure B), and after completion of 40 treatments of hyperbaric oxygen (subfigure C).

He denied any constitutional symptoms, oral lesions, nail changes, urinary symptoms, bowel symptoms, musculoskeletal symptoms, trauma to the area, or previous sexually transmitted diseases. On initial examination of the genitals, there was a violaceous appearance of the glans, a small erosion on the ventral coronal sulcus with overlying serosanguinous exudate, incidental angiokeratomas near the scrotum, and scrotal edema (Figure 1A). Psoriatic lesions was present on his extremities which had been present for a few months.

## Diagnostic Focus and Assessment

His penile pain was addressed by the nephrology, urology, and dermatology services. Calciphylaxis was considered early but the inconspicuous appearance of the lesion, recent initiation of dialysis, absence of other foci, and atypical location made the diagnosis unlikely. Urology initially suspected infection. Skin cultures returned positive for light growth of skin flora. Dermatology was concerned for multiple entities, given the immense pain of the lesion: infection, fixed drug eruption, neoplasm, or ischemic process. A viral swab for HSV/VZV and serologies for CMV/EBV were negative. A 2-mm punch biopsy revealed a fragment of granulation tissue consistent with biopsy from an ulcer bed, scant epithelial cells with reactive features, no evidence of viral cytopathic events, an immunostain negative for p16, and a stain negative for spirochetes. The patient was discharged with early follow-up to see dermatology and urology.

**Figure 2.**
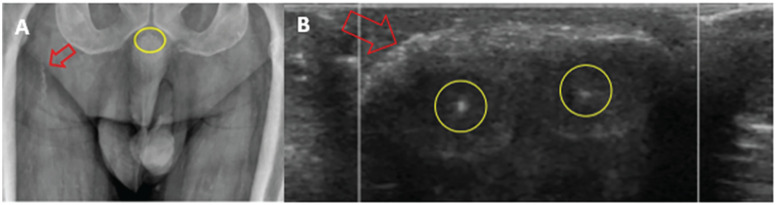
Imaging findings supportive of the diagnosis of calciphylaxis. Plain radiograph of pelvis (subfigure 2A) revealing calcifications in the vascular structures at the base of the penis (yellow circle) and calcified vessel in upper right anterior thigh (red arrow). US of glans penis (subfigure 2B) revealing calcification of both penile arteries (yellow circles) and dense curvilinear calcification of the glans penis (red arrow).

The patient represented in September with worsening of their penile lesion (Figure 1B). The urology team obtained a second 2-mm punch biopsy which revealed microthrombi in the deep vessels with resultant ischemic necrosis of the epithelium and submucosal connective tissue. A Gram stain revealed gram-positive cocci with no vital reaction. Grocott and PAS stains showed no fungal organisms. Viral cytopathic changes were absent and immunohistochemical stains for CMV were negative. There was no evidence of a primary vasculitis. Direct immunofluorescence of the sample was unremarkable. Plain radiography detected calcium deposition in the vascular structures of the base of the penis (Figure 2A). Ultrasound revealed vascular calcifications along both penile arteries and superficial dense curvilinear calcification of the glans penis (Figure 2B). These findings were sufficient to generate reasonable certainty in the diagnosis of calciphylaxis.

## Therapeutic Focus and Assessment

After the diagnosis of calciphylaxis was made the patient was transitioned from PD to thrice weekly intermittent hemodialysis (IHD) and started on hyperbaric oxygen therapy (HBOT). An Exceptional Access Program (EAP) request for use of sodium thiosulphate (STS) was submitted and starting September 25, 2020, 25 g of STS has been administered during each IHD session with a tentative stop date of March 24, 2021. HBOT occurred daily for 40 days. The patient underwent 90 minutes per session at 2.5 atmospheres absolute (ATA) of hyperbaric oxygen in 30-minute subsessions separated by 10-minute intervals at room air. His calcium carbonate was replaced with a noncalcium containing phosphate-binder, Sevelamer.

## Follow-Up and Outcomes

A 49-year-old man with a history of ESRD presented with sudden onset penile pain and an ambiguous violaceous discoloration on the glans of his penis. His presentation occurred just prior to the initiation of dialysis. Initial biopsy was inconclusive, and the patient was discharged with pain management options as well as follow-up with nephrology, urology, and dermatology. The patient represented prior to any scheduled follow-up appointments with worsening of his penile lesion and an area of necrosis on the glans of his penis. Given his risk factors and negative evaluation for alternative causes the suspicion for calciphylaxis was high. Repeat punch biopsy was inconclusive, and there were no other lesions to investigate. Plain radiograph of the pelvis demonstrated calcifications at the base of the penile arteries and penile ultrasound revealed vascular calcifications along both penile arteries and superficial dense curvilinear calcification of the glans penis. A diagnosis of isolated penile calciphylaxis was made. The patient was transitioned to IHD with STS, started on HBOT, and had their calcium carbonate switched to Sevelamer.

**Figure 3.**
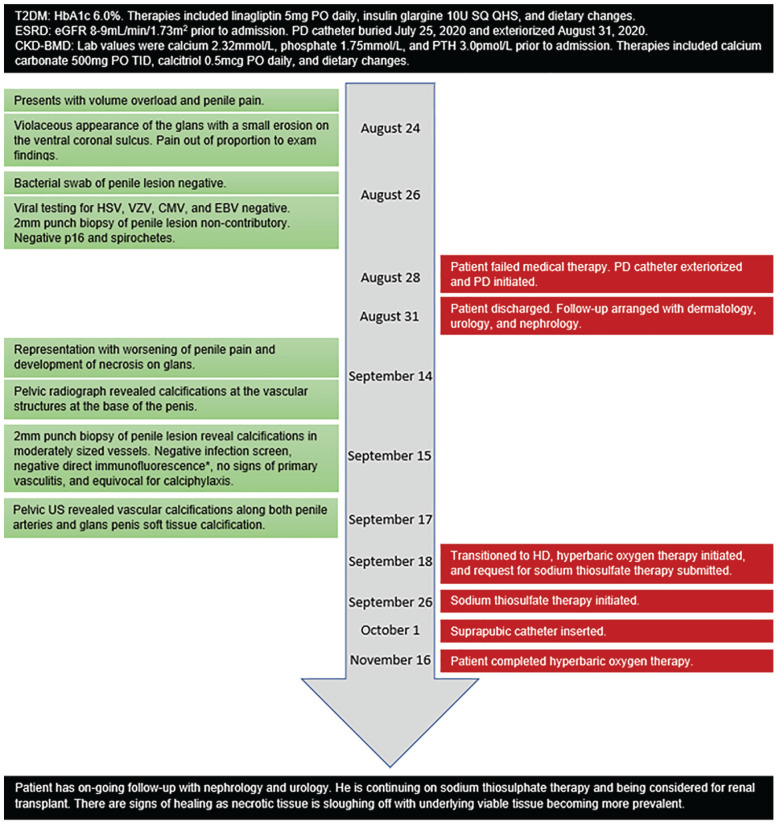
Timeline of clinical course.

The patient completed HBOT without side-effects except for moderate myopia. His vision is expected to revert to normal within a few months. At the end of his HBOT, his wound contained granulation tissue with sloughing off of some overlying necrotic tissue (Figure 1C). There has been no self-amputation or planned surgical intervention. A suprapubic catheter has been inserted due to outflow obstruction associated with necrosis. The patient is overall satisfied with the progress they have made. A summary of relevant events in in the patient’s clinical course are illustrated in Figure 3. They continue to have follow-up with the nephrology, dermatology, and urology services.

## Discussion

Calciphylaxis is a syndrome characterized by occlusion of microvasculature in primarily subcutaneous adipose tissue and dermis secondary to vascular calcification causing ischemic necrosis.^1^ Calciphylaxis can be either uremic in the context of ESRD (calcific uremia arteriolopathy or CUA) or nonuremic.^1,2^ Elevated PTH level at the start of dialysis, primary hyperparathyroidism, administration of recombinant PTH, inadequate HD, PD, younger age, female gender, vitamin K antagonist use or deficiency, and elevations in calcium or phosphate are associated with higher risk of calciphylaxis.^1-3,5^ Central lesions affecting the abdomen and thighs are most likely in obese patients and patients with ESRD.^1,5^ Peripheral lesions account for approximately 50% of lesions in those without ESRD.^7^ Involvement of other tissues has been well-described in various locations including lung, brain, muscle, intestine, and eye.^8^ Prognosis is poor in patients with ESRD and calciphylaxis, with a mortality rate of 45% to 80% after 1 year.^5,9^ The most common cause of death is sepsis from a wound.^1,2^ The need for amputations or debridement is common.^1,2^

Calcium and phosphate regulation is mediated by PTH, FGF-23, and 1,25-Vitamin D. In CKD, the impaired excretion of phosphate and limited production of 1,25-Vitamin D leads to hyperparathyroidism, high FGF-23, hyperphosphatemia, and hypocalcaemia. Toxin accumulation and inflammatory mediators induce transdifferentiation of contractile vascular smooth muscle cells (VSMC) into osteochondrogenic VSMC by disturbing the balance of procalcifying and anticalciphying mediators.^1,8^ After transdifferentiation, an imbalance of calcification promoters and inhibitors drives arteriolar medial wall mineralization.^1,8^ Arteriolar medial calcification and intimal fibrosis lead to ischemia that perpetuates endothelial dysfunction and thrombus formation resulting in complete occlusion.^1,2,7^

A high index of suspicion is required for calciphylaxis as lesions can be clinically diverse.^8,10,11^ Lesions typically present as indurated subcutaneous plaques that are exquisitely tender and often have overlying livedo reticularis.^10,12^ Pain is classically out-of-proportion to physical examination and may present prior to skin lesions.^10,11^ Lesions will progress to stellate, malodorous ulcers, with black eschars.^11^ Skin biopsy is the gold standard for diagnosis, but its role is debated due to the risk of precipitating new lesions.^1^ Samples should undergo specialized staining for calcification.^1^ Positive biopsies demonstrate stippled calcifications involving the capillaries, fibrointimal hyperplasia, or thrombi in microvasculature.^13^ Imaging findings are not diagnostic of calciphylaxis but can support the diagnosis.^1,2^ Computed tomography (CT), plain radiography, ultrasound, mammography, and bone scintigraphy have been used to diagnose calciphylaxis.

Bone scintigraphy has been utilized in the diagnosis of calciphylaxis but has limited data in the setting of penile calciphylaxis.^4^ The role of bone scintigraphy in calciphylaxis includes diagnosis, prognostication, and monitoring response to therapy.^14^ The sensitivity and specificity in the diagnosis of calciphylaxis has been reported as 89% and 97%, respectively, in a recent retrospective analysis but a sensitivity of up to 97% has been reported.^14,15^

Presence of vascular calcification on plain radiograph was found to be 90% sensitive for diagnosing calciphylaxis.^6^ A netlike pattern of calcification conferred an odds ratio of 9.4 and specificity of 89.9%.^6,16^ In one study, at least moderate calcification was detected in all 4 patients with calciphylaxis who received a plain radiograph.^16^

Ultrasound demonstrates calcified vessels as having hyperechoic walls and posterior shadowing when performing a Doppler ultrasound examination.^16^ Calcium deposits appear similarly in soft-tissue and have been demonstrated on point-of-care-ultrasound (POCUS) to support a diagnosis of calciphylaxis.^17^ Doppler ultrasound has demonstrated to be a useful tool in guiding surgical intervention in penile calciphylaxis by quantifying arterial and venous blood flow.^18,19^ Penile ultrasound can be useful even in the presence of normal macrovascular perfusion by demonstrating diffuse penile calcifications.^20^

Studies that have investigated the management of calciphylaxis are predominantly based on observational data and there is a need for randomized control trials (RCTs). The treatment of calciphylaxis is primarily supportive with focus on analgesia and wound management.^1^ Other medications that are implicated in the development of calciphylaxis, such as warfarin, should be avoided.^21^ Nutritional status should be optimized and the prophylactic use of antibiotics is not recommended.^1^ Debridement and amputation are often necessary for necrotic or gangrenous lesions.^1,21^ An aberrant calcium-phosphate-parathyroid hormone axis should be aggressively corrected, and calcium-containing phosphate binders should be avoided.^1,21^ Parathyroidectomy is generally reserved for refractory cases of hyperparathyroidism unresponsive to calcimimetics, selective vitamin D analogues, and noncalcium phosphate binders.^21^ Those who are receiving PD should be switched to HD and consideration should be given to 4 times weekly HD instead of 3 times weekly HD with low calcium containing dialysate.^1,21^ Wound healing may be assisted by HBOT. A retrospective study including 34 patients with predominately peripheral lesions resulted in complete healing in half of the patients after 44 sessions.^22^ Pooled data from a recent systematic review which included 131 patients treated with HBOT did not demonstrate benefit in wound progression, need for amputation, or mortality; however, there was wide variability in the number of sessions patients received with a mean of 40.8 and a standard deviation of 24.1.^22^

Adjunctive pharmacologic measures include bisphosphonates and STS.^1,21,23^ Bisphosphonates are thought to inhibit calcium crystallization and prevent hydroxyapatite formation in vascular walls, but their exact mechanism of action is unknown, they must be used cautiously in CKD, and they offered no statistically significant pooled risk ratio reduction in mortality from data including 623 patients treated with bisphosphonates.^22^ STS is thought to chelate calcium by forming the relatively water-soluble calcium thiosulphate, act as an antioxidant, induce vasodilation, and antagonize the ability of adipocytes to induce calcification of VSMC.^1,23^ No statistically significant difference in wound progression, need for amputation, or mortality was reported in a metanalysis which included 431 patients who received STS.^22^ A separate retrospective case series investigating 172 maintenance hemodialysis patients who received STS for calciphylaxis reported a 1-year mortality of 35% which the authors argued was less than the 1-year mortality of 55% reported in literature in those who did not receive STS.^23^ Two RCTs investigating STS therapy in calciphylaxis are currently pending publication (current controlled trials number, ISRCTN73380053; and ClinicalTrials.gov number, NCT03150420).

Penile calciphylaxis is a rare but well-described phenomenon in literature with approximately 50 cases supported with less than half of these presenting with isolated penile calciphylaxis and only 4% occuring in the predialysis stage of CKD.^20^ The differential of penile calciphylaxis includes infections (primarily sexually transmitted infections), primary neoplasms, trauma, fixed drug eruptions, cutaneous Crohn’s disease, pyoderma gangrenosum, erosive lichen planus, and contact dermatitis.^24^ The diagnosis of penile calciphylaxis is exceeding difficult because of the atypical location and often ambiguous presentation. Prompt recognition and diagnosis is paramount for salvaging the appendage. The biopsies in our case were nondiagnostic but plain radiograph and US findings were consistent with calciphylaxis. These tests created reasonable certainty in the diagnosis which permitted the timely initiation of STS and HBOT. This case study should reinforce the high level of vigilance required of clinicians to promptly recognize calciphylaxis and advocate for the currently underutilized methods of US and plain radiographs as widely available, inexpensive, noninvasive adjunctive investigations in the diagnosis of calciphylaxis.
